# Effects of gut microbiome-targeted therapies on cardiometabolic outcomes in children and adolescents: A protocol for systematic review and meta-analysis

**DOI:** 10.1097/MD.0000000000021612

**Published:** 2020-07-31

**Authors:** Liyuan Yan, Minghan Wang, Jingjing Chen, Xin Zhao, Haipeng Wang

**Affiliations:** Department of Cardiology, the First Affiliated Hospital of Soochow University, Suzhou, Jiangsu, China.

**Keywords:** adolescent, cardiovascular risk factors, child, meta-analysis, probiotic, synbiotic

## Abstract

**Background:**

Emerging evidence indicates the role of gut microbiota in the development of cardiovascular diseases. Thus, gut microbiota is increasingly recognized as a potential therapeutic target of cardiovascular disease. However, the effects of gut microbiome-targeted therapies on cardiometabolic outcomes in children and adolescents remain unclear.

**Methods:**

We plan to perform a systematic search from PubMed, EMBASE, Cochrane Central Register of Controlled Trials, and Web of Science. Two authors will independently select the relevant studies and extract data according to a previously defined data extraction sheet. We will use the Stata 14.0 statistical software and RevMan V.5.3 software to conduct data analyses.

**Results and conclusion:**

The results of this study will be published in a peer-reviewed journal and provide more evidence for the application of gut microbiome-targeted therapies in children and adolescents for the intervention of cardiovascular risk factors in clinical practice.

**Protocol registration number:**

INPLASY202060050.

## 1 Introduction

Cardiovascular disease is the leading cause of non-communicable disease deaths worldwide, and deaths resulting from cardiovascular disease rose by 12.5% from 2005 to 2015.^[1]^ Abnormal levels of cardiometabolic outcomes such as serum lipids, blood pressure, and glucose homeostasis variables in childhood persist into adulthood and also contribute to the occurrence of type 2 diabetes and cardiovascular disease in the future.^[2–6]^ Therefore, it is necessary to prevent the occurrence of cardiovascular disease from childhood. Emerging evidence indicates that gut microbiota and gut microbiota-derived metabolites are correlated to cardiovascular disease, which indicates that gut microbiota may serve as a potential therapeutic target for cardiovascular disease.^[7–9]^ Pro-/synbiotic supplementation, a gut microbiota-targeted intervention, attracts increasing attention in recent years for its potential benefits for people with cardiovascular disease or cardiovascular risk factors.^[10]^ Probiotics are defined as live microorganisms that are of benefit to the host when administered in enough amounts and duration.^[11]^ Prebiotics, such as fructooligosaccharides and inulin, are non-digestible carbohydrates that are utilized by gut microbiome for fermentation and play a role in inducing the metabolism and growth of microorganisms.^[11]^ Synbiotics are a mixture of probiotics and prebiotics.^[11]^ There have been several studies investigating the effects of gut microbiome-targeted therapies on cardiometabolic outcomes in children and adolescents.^[12–22]^ However, the results of previous trials are inconsistent. A meta-analysis published in 2019 indicates that pro-/synbiotic supplements have no beneficial effects on fasting blood glucose and blood lipids in overweight or obese children and adolescents.^[23]^ However, in their study, only overweight or obese children were included, and only five cardiovascular risk factors were analyzed. Therefore, we plan to systematically and comprehensively evaluate the effects of gut microbiome-targeted therapies (pro-/synbiotic supplements) on cardiometabolic outcomes in children and adolescents.

## 2 Methods

### 2.1 Study design and registration

The protocol for this systematic review was registered on INPLASY (INPLASY202060050) and is available in full on the inplasy.com (https://doi.org/10.37766/inplasy2020.6.0050). We will perform the study in full accordance with the Preferred Reporting Items for Systematic Reviews and Meta-Analyses Protocol 2015 statement.^[24]^

### 2.2 Eligibility criteria

#### 2.2.1 Types of studies.

We plan to include only randomized controlled trials that investigate the effects of gut microbiome-targeted therapies on cardiometabolic outcomes in children and adolescents. Animal studies, case reports, observational studies, noncontrolled trials, study protocols, and duplicate publications will not be included in this study.

#### 2.2.2 Types of participants

Participants were 0 to ≤18 years of age at baseline. Studies were excluded if they included malnourished participants.

#### 2.2.3 Types of interventions and controls

In the experimental group, all participants received probiotics or synbiotics at any dose, duration, and route of administration, given in combination or separately. In the control group, all participants received placebos, no intervention, or other interventions, except probiotics or synbiotics.

#### 2.2.4 Types of outcomes

The study outcomes include total cholesterol, high-density lipoprotein cholesterol, low-density lipoprotein cholesterol, triglyceride, systolic blood pressure, diastolic blood pressure, fasting blood glucose, glycated hemoglobin, insulin, and homeostatic model assessment of insulin resistance.

### 2.3 Search strategy

We plan to systematically search the following databases from inception to the present: PubMed, EMBASE, Cochrane Central Register of Controlled Trials, and Web of Science. There will be no limitation for language and date of publication. The search strategy for PubMed is shown in Table 1. We will use similar search strategies to identify relevant articles in other electronic databases. To include every potential article, we will also search grey articles, such as references of relevant reviews, and conference abstracts.

**Table 1:**
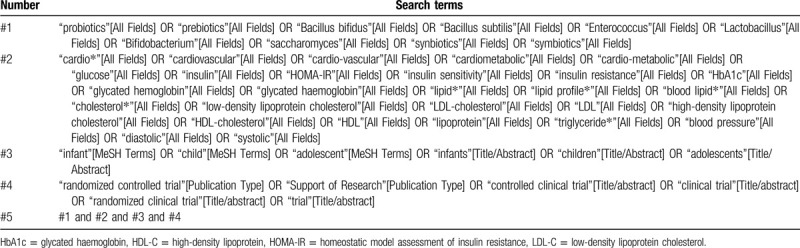
Search strategy of PubMed.

### 2.4 Study selection and management

**Figure 1:**
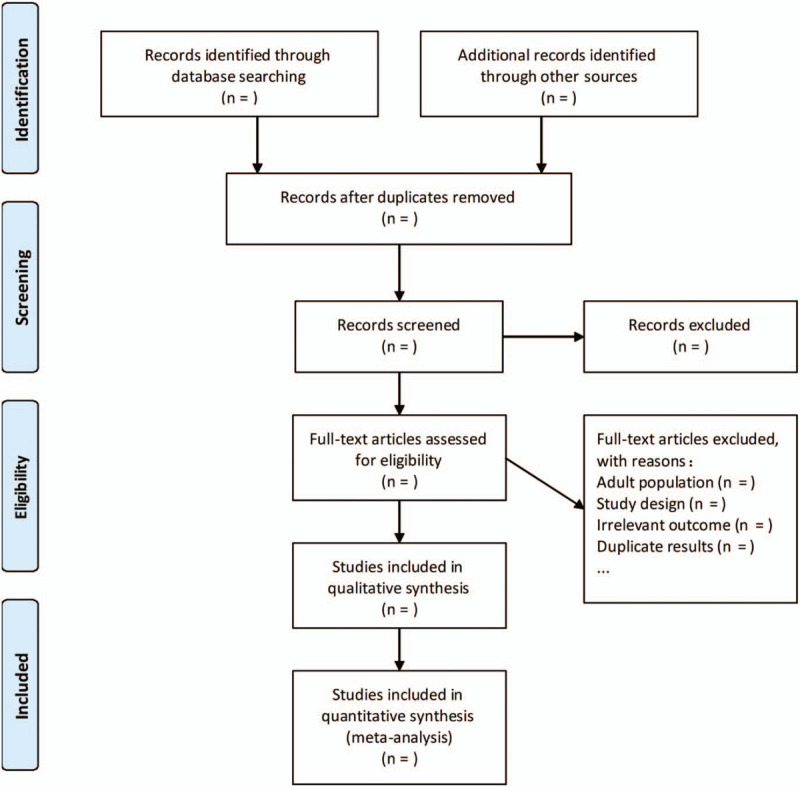
The PRISMA flow diagram of the study selection and exclusion. PRISMA = preferred reporting items for systematic reviews and meta-analyses.

Two independent authors (Liyuan Yan and Haipeng Wang) will perform the selection process for eligible studies by using the Endnote X9 software (Thomson Research Soft, Stanford, Connecticut). Firstly, we will read the title and abstract to select appropriate studies and remove irrelevant studies. Then, we will read the full text of all remaining articles to determine whether a trial should be included. Any disagreement between the two authors will be discussed with a third author (Xin Zhao). The selection details will be shown in the Preferred Reporting Items for Systematic Reviews and Meta-Analyses compliant flow diagram (Fig. 1).

### 2.5 Data extraction and management

Two authors (Liyuan Yan and Haipeng Wang) will independently extract data from selected articles based on a previously defined data extraction sheet. The following data will be extracted from selected articles: first author, publication year, country, target population, sample size, age, gender distribution, intervention details, study design, intervention duration, outcome measurements, and any other relevant information.

### 2.6 Dealing with missing data

We will contact the authors of selected articles to obtain missing data and clarify unclear data.

### 2.7 Risk of bias assessment

Two authors (Minghan Wang and Jingjing Chen) will be responsible for evaluating the quality of selected studies by using the Cochrane Collaboration's tool.^[25]^ The Cochrane Collaboration's tool include types of biases as follows:

(1) selection bias (random sequence generation and allocation concealment);
(2) performance bias (blinding of participants and personnel);
(3) detection bias (blinding of outcome assessment);
(4) attrition bias (incomplete outcome data);
(5) reporting bias (selective reporting); and
(6) other bias.

The risk of bias for each item will be classified as high risk, low risk, or unclear risk. Any disagreement between the 2 authors will be discussed with a third author (Xin Zhao).

### 2.8 Strategy of data synthesis

We plan to conduct the meta-analysis by using the Stata 14.0 (Stata Corp, College Station, TX) statistical software and RevMan V.5.3 software (Nordic Cochran Centre, Copenhagen, Denmark). Continuous data will be expressed using standardized mean difference or mean difference and 95% confidence intervals. Cochrane *Q*-test and *I*^2^ statistics will be used to assess the inter-study heterogeneity. If *I*^2^ ≤50%, a fixed-effects model will be used to combined results of included studies. A random-effects model will be used to calculate the outcome when *I*^2^ >50%.

### 2.9 Assessment of heterogeneity

#### 2.9.1 Subgroup analysis

When there is significant heterogeneity between studies, we will perform subgroup analysis based on types of gut microbiome-targeted therapies, intervention duration, and target population.

#### 2.9.2 Sensitivity analysis

We will perform sensitivity analysis to test the stability of each outcome result by removing studies with insufficient data and a high risk of bias.

### 2.10 Publication bias analyses

Publication bias is always an inevitable problem when performing a meta-analysis. When there are at least ten articles concerning the same outcome variable left, we will perform Egger regression asymmetry test and Begg rank correlation test to evaluate publication bias.

### 2.11 Quality of evidence

We will use the Grading of Recommendations Assessment, Development and Evaluation method to evaluate the quality of evidence of the present systematic review and meta-analysis.

## 3 Discussion

There have been several randomized controlled trials aiming to evaluate the effects of pro-/synbiotic supplements on cardiometabolic outcomes (blood pressure, serum lipids, and glucose homeostasis variables) in children and adolescents with inconsistent results.^[12–22]^ We plan to provide a summary of the current evidence concerning the effects of gut microbiome-targeted therapies on cardiovascular risk factors in children and adolescents. The results of our study will be published in a peer-reviewed journal and provide more substantial evidence in clinical practice.

## 4 Ethics and dissemination

The approval from the ethics committee is not needed because our study is based on previously published trials. The results of this study are going to be published in a peer-reviewed journal after we finish the systematic review and meta-analysis.

## Author contributions

**Conceptualization:** Liyuan Yan.

**Data curation:** Liyuan Yan, Haipeng Wang, Xin Zhao.

**Formal analysis:** Liyuan Yan, Minghan Wang, Jingjing Chen.

**Methodology:** Liyuan Yan.

**Project administration:** Liyuan Yan, Xin Zhao.

**Resources:** Haipeng Wang, Xin Zhao.

**Software:** Liyuan Yan, Minghan Wang, Jingjing Chen.

**Supervision:** Haipeng Wang, Xin Zhao.

**Writing – original draft:** Liyuan Yan.

**Writing – review & editing:** Haipeng Wang, Xin Zhao.
